# High-Titer Neutralizing Antibodies against the SARS-CoV-2 Delta Variant Induced by Alhydroxyquim-II-Adjuvanted Trimeric Spike Antigens

**DOI:** 10.1128/spectrum.01695-21

**Published:** 2022-02-16

**Authors:** Claudio Counoupas, Paco Pino, Alberto O. Stella, Caroline Ashley, Hannah Lukeman, Nayan D. Bhattacharyya, Takuya Tada, Stephanie Anchisi, Charles Metayer, Jacopo Martinis, Anupriya Aggarwal, Belinda M. Dcosta, Warwick J. Britton, Joeri Kint, Maria J. Wurm, Nathaniel R. Landau, Megan Steain, Stuart G. Turville, Florian M. Wurm, Sunil A. David, James A. Triccas

**Affiliations:** a School of Medical Sciences, Faculty of Medicine and Health, The University of Sydney, Camperdown, New South Wales, Australia; b Tuberculosis Research Program, Centenary Institute, Sydney, New South Wales, Australia; c ExcellGene SA, Monthey, Switzerland; d Kirby Institute, University of New South Wales, Sydney, New South Wales, Australia; e Department of Microbiology, NYU Grossman School of Medicine, New York, New York, USA; f Life Science Faculty, Swiss Federal Institute of Technology Lausanne (EPFL), Lausanne, Switzerland; g Virovax, Lawrence, Kansas, USA; h Sydney Institute for Infectious Diseases and the Charles Perkins Centre, The University of Sydney, Camperdown, New South Wales, Australia; Texas A&M University

**Keywords:** COVID-19, vaccination, subunit vaccine, variant, adjuvant, immune response

## Abstract

Global control of COVID-19 will require the deployment of vaccines capable of inducing long-term protective immunity against SARS-CoV-2 variants. In this report, we describe an adjuvanted subunit candidate vaccine that affords elevated, sustained, and cross-variant SARS-CoV-2 neutralizing antibodies (NAbs) in multiple animal models. Alhydroxiquim-II is a Toll-Like Receptor (TLR) 7/8 small-molecule agonist chemisorbed on aluminum hydroxide (Alhydrogel). Vaccination with Alhydroxiquim-II combined with a stabilized, trimeric form of the SARS-CoV-2 spike protein (termed CoVac-II) resulted in high-titer NAbs in mice, with no decay in responses over an 8-month period. NAbs from sera of CoVac-II-immunized mice, horses and rabbits were broadly neutralizing against SARS-CoV-2 variants. Boosting long-term CoVac-II-immunized mice with adjuvanted spike protein from the Beta variant markedly increased levels of NAb titers against multiple SARS-CoV-2 variants; notably, high titers against the Delta variant were observed. These data strongly support the clinical assessment of Alhydroxiquim-II-adjuvanted spike proteins to protect against SARS-CoV-2 variants of concern.

**IMPORTANCE** There is an urgent need for next-generation COVID-19 vaccines that are safe, demonstrate high protective efficacy against SARS-CoV-2 variants and can be manufactured at scale. We describe a vaccine candidate (CoVac-II) that is based on stabilized, trimeric spike antigen produced in an optimized, scalable and chemically defined production process. CoVac-II demonstrates strong and persistent immunity after vaccination of mice, and is highly immunogenic in multiple animal models, including rabbits and horses. We further show that prior immunity can be boosted using a recombinant spike antigen from the Beta variant; importantly, plasma from boosted mice effectively neutralize multiple SARS-CoV-2 variants *in vitro*, including Delta. The strong humoral and Th1-biased immunogenicity of CoVac-II is driven by use of Alhydroxiquim-II (AHQ-II), the first adjuvant in an authorized vaccine that acts through the dual Toll-like receptor (TLR)7 and TLR8 pathways, as part of the Covaxin vaccine. Our data suggest AHQ-II/spike protein combinations could constitute safe, affordable, and mass-manufacturable COVID-19 vaccines for global distribution.

Coronavirus Disease 2019 (COVID-19) vaccines have had a remarkable impact on controlling the pandemic in high and middle-income countries. However, global access to affordable COVID-19 vaccines remains a critical issue (1). Neutralizing antibodies (NAbs) are considered the key determinant of SARS-CoV-2 protective immunity (2, 3), yet in both natural infection and vaccination the levels of NAbs decay over time (4, 5). This issue is compounded by the emergence of severe acute respiratory syndrome associated Coronavirus 2 (SARS-CoV-2) variants circulating that show partial resistance to current vaccines (6, 7), highlighting the need for next-generation vaccines that display strong and persistent immunity.

Subunit vaccines represent a safe and affordable platform for COVID-19 vaccines, with positive outcomes for some candidates from clinical trials (8, 9). To develop a subunit vaccine that is suitable for global distribution, large-scale antigen manufacture and selection of adjuvants are critical issues. To address these, we used trimeric SARS-CoV-2 spike protein stabilized by 2 consecutive proline substitutions in the S2 subunit, produced with a fully scalable, chemically defined and GMP-compliant production process (10). To create the CoVac-II vaccine candidate, trimeric spike protein was combined with Alhydroxiquim-II (AHQ-II); a small-molecule imidazoquinoline Toll-Like Receptor (TLR) 7/8 agonist chemisorbed to Alhydrogel (11, 12). AHQ-II induces Th1-biased immunity and is the adjuvant used in the Covaxin COVID-19 vaccine, which has received emergency use approval in multiple countries (13). Importantly, recombinant spike trimer expressed and purified from Chinese hamster ovary (CHO) cells was stable when stored at multiple temperatures and resistant to repeated freeze-thaw cycles (Fig. S1). Following vaccination of mice with CoVac-II (Ancestral Wuhan SARS-CoV-2 spike antigen formulated in AHQ-II, Fig. 1A), high-titer NAbs were apparent in plasma as early as 2 weeks after the first immunization (Fig. 1B). NAb titers further increased after boosting and remained at high levels over the course of the 252 days follow-up period postvaccination. This corresponds with the excellent stability of both the spike protein (Fig. S1) and adjuvant used; AHQ-II is stable for at least 1 year at room temperature (not shown). Compared to spike antigen alone, AHQ-II increased NAb titers by approximately 1000-fold (*P* < 0.01). This increase compares favorably to the effect seen with other adjuvants used in SARS-CoV-2 subunit vaccines; e.g., the approximately 10-fold increase observed with Matrix-M (saponin-based nanoparticles) or the approximately 500-fold increase observed with the AS03 adjuvant (squalene-based oil-in-water emulsion) (14, 15). We observed that when trimeric spike antigen was formulated in Alhydrogel (SpK^Alum^) it induced NAb titers in mice above those elicited by spike protein alone; however, titers were approximately 100-fold lower at day 252 compared to those from CoVac-II-vaccinated animals (*P* < 0.01) (Fig. 1B).

**FIG 1:**
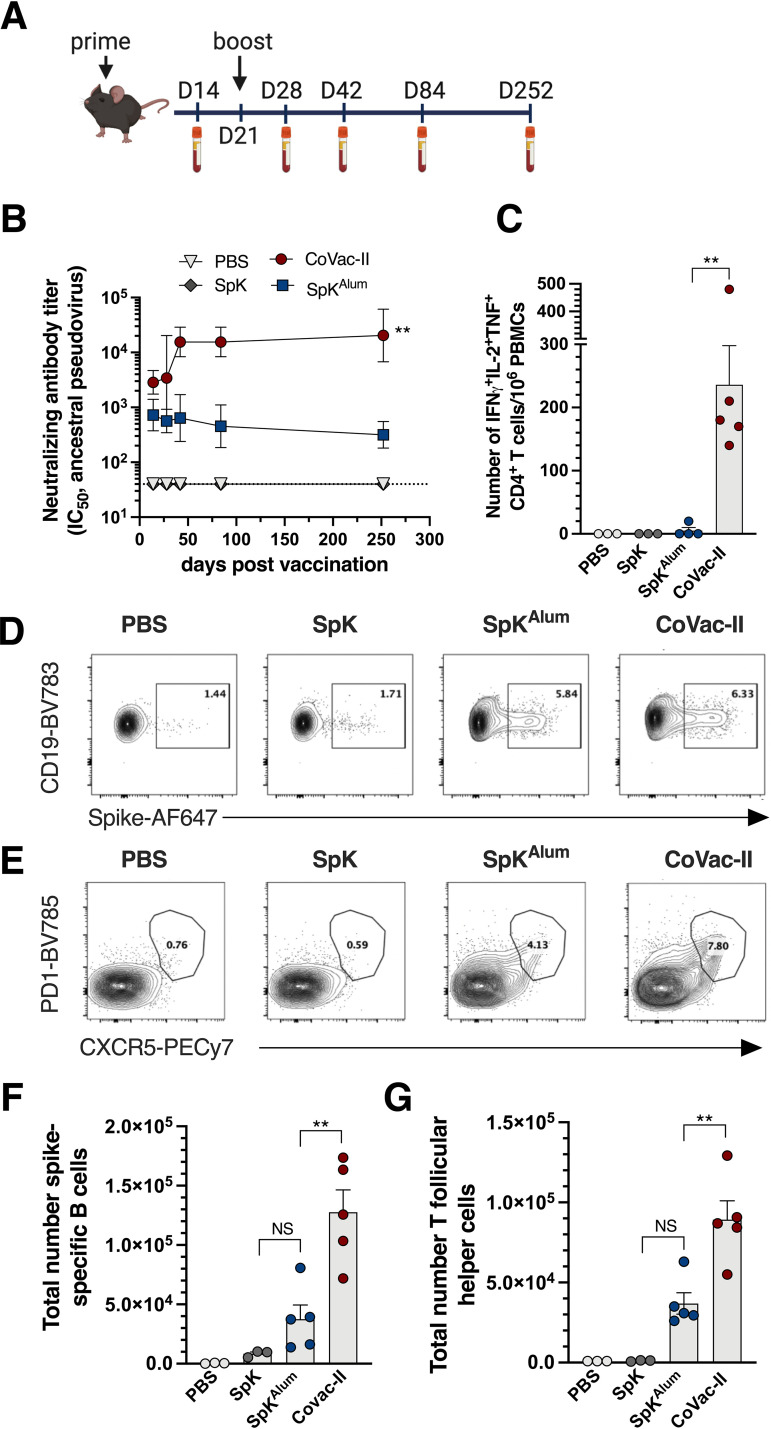
Sustained neutralizing antibody titers and generation of multifunctional CD4^+^ T cells responses after vaccination with Alhydroxiquim-II-adjuvanted spike antigen. (A) C57BL/6 mice (*n* = 4 to 5) were vaccinated s.c at day 1 and day 21 with PBS, SpK (5 μg spike), SpK^Alum^ (5 μg spike/100 μg Alhydrogel) or CoVac-II (5 μg spike/100 μg AHQ-II). (B) Neutralizing antibody (NAb) titers (IC_50_) in plasma were determined using ancestral spike-pseudotyped virus. Dotted line shows the limit of detection. No response was seen with adjuvant alone (not shown). (C) PBMCs taken 1 week post-boost were restimulated with 5 μg/mL of SARS-CoV-2 spike protein and the number of cytokine-expressing CD4^+^ T cells determined by flow cytometry. (D) G. C57BL/6 mice were vaccinated as in A and at 7 days the frequency of spike-specific B cells gated based on their expression of both CD19^+^ and MHCII^+^ (D) or T follicular helper T cells (Tfh) gated based on their expression of both chemokine receptor 5 (CXCR5^+^) and programmed cell death protein 1 (PD1^+^) (E) was determined by flow cytometry. The total number of spike^+^ B cells (F) and Tfh cells (G) is also shown. Data presented as geometric mean ± geometric SD (B) or mean ± SEM (C, F, G). Significant differences between groups were determined by one-way ANOVA; **P < 0.01. PE-Cy7: Phycoerythrin Cyanin 7; AF647: Alexafluor647; BV785: Brilliant Violet785. Panel A created with BioRender.com.

To determine the pattern of T cell immunity induced by the CoVac-II vaccine, we assessed the level of spike-specific multifunctional CD4^+^ T cells in peripheral blood mononuclear cells (PBMC) from vaccinated mice. High frequency of CD4^+^IFN^+^IL-2^+^TNF^+^ were seen after CoVac-II delivery, that were absent from mice vaccinated with spike antigen alone or SpK^Alum^ (Fig. 1C). This corresponds with the Th1-polarising effect of AHQ-II when delivered with inactivated SARS-CoV-2 in animal models and humans (16, 17). We also noted a significant correlation between NAb titers and the level of multifunctional CD4^+^ T cells produced in individual mice (Fig. S2). To further dissect vaccine-induced immunity, we examined the cellular make-up of the draining lymph nodes 7 days after vaccination. Both SpK^Alum^ and CoVac-II induced expansion of spike-specific B cells (CD19^+^MHCII^+^; Fig. 1D) and T follicular helper cell (Tfh) cells (CD4^+^CXCR5^+^PD1^+^; Fig. 1E). The total numbers of antigen specific B cells (Fig. 1F), and Tfh cells (Fig. 1G) were significantly increased in CoVac-II-vaccinated mice compared to immunization with SpK^Alum^. Thus, delivery of trimeric spike antigen with AHQ-II results in strong B and T cell anti-SARS-CoV-2 immune response.

The ability of CoVac-II-induced antibodies to neutralize SARS-CoV-2 variants of concern (VOCs) was examined using isolates of ancestral (Wuhan), as well as Alpha and Beta variants (4). All plasma samples from CoVac-II-immunized mice neutralized both Alpha and Beta VOCs however titers were reduced for Beta compared to the ancestral virus (8-fold reduction), which is a similar reduction as seen for other COVID-19 vaccines (Fig. 2A) (7). Nab titers using plasma from SpK^Alum^-vaccinated mice were reduced to the limit of detection against the Beta VOC (Fig. 2B). Cross-species neutralization of VOCs was apparent after immunization of rabbits (Fig. 2C) or horses (Fig. 2D) with CoVac-II. NAb titers were maintained against the Alpha variant in both species compared to ancestral virus, and high NAb titers were observed against Beta VOC in rabbits (4.7-fold reduction compared to ancestral) and horses (2.7-fold). Thus, AHQ-II can adjuvant vaccine immunogenicity across multiple animal models, adding to its already proven immunogenicity in humans as part of the Covaxin vaccine (13).

**FIG 2:**
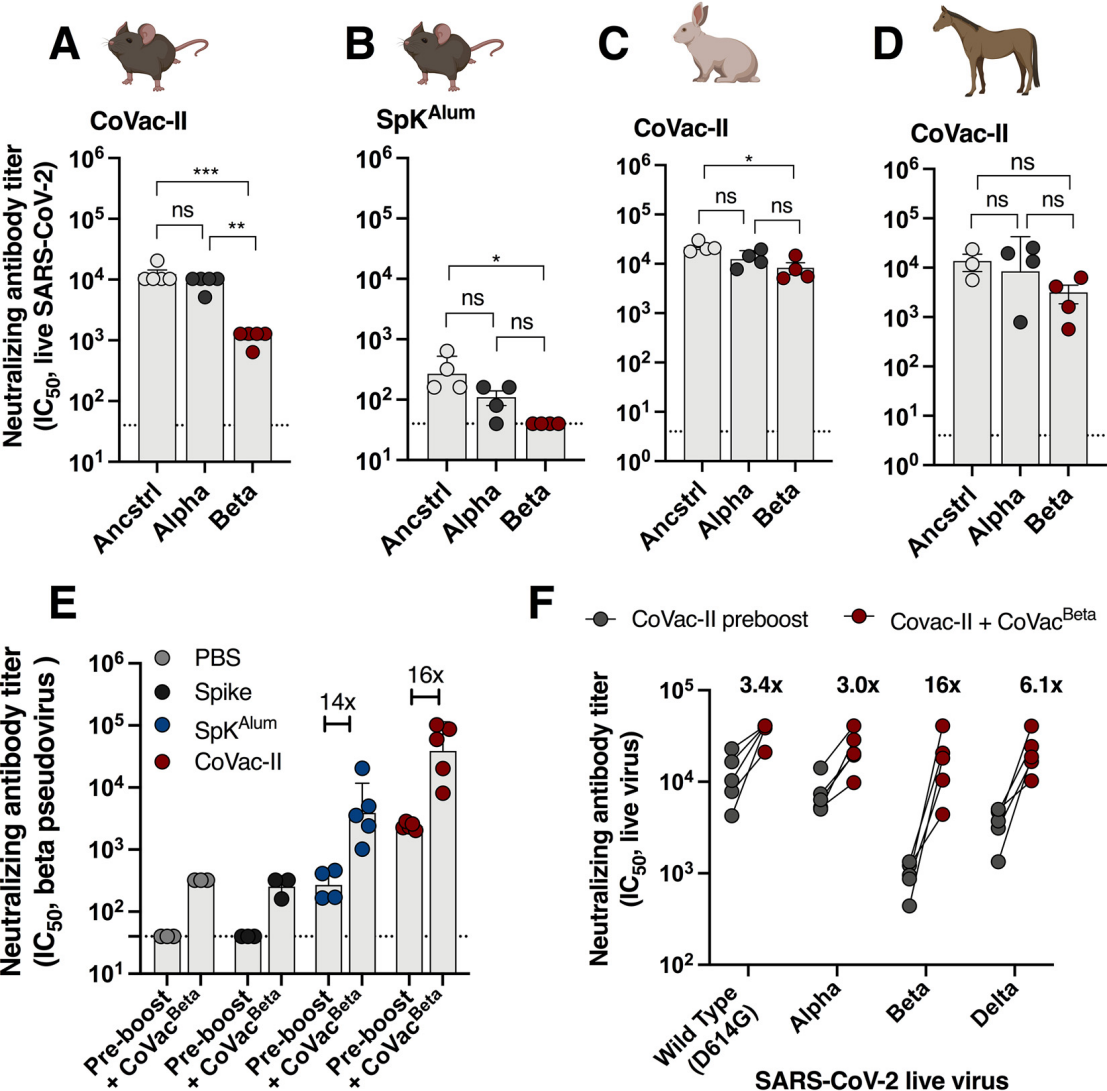
Alhydroxiquim-II-adjuvanted vaccines afford cross-species neutralization of variants of concern, that is augmented by a variant-specific booster vaccine. C57BL/6 mice (*n* = 4 to 5) were vaccinated as in Fig. 1 and 3 weeks postvaccination plasma from CoVac-II (A) or SpK^Alum^ (B) groups were tested for neutralizing activity against live SARS-CoV-2 infection of Vero E6 cells (ancstrl = ancestral virus). (C) Rabbits (*n* = 3) were immunized i.m. twice with CoVac-II (5 μg ancestral spike/200 μg AHQ-II) and NAb titers against live SARS-CoV-2 viruses determined. (D) Horses (*n* = 3) were immunized i.m. twice with CoVac-II (20 μg ancestral spike/500 μg AHQ-II) and NAb titers against live SARS-CoV-2 viruses determined. (E) Mice vaccinated 252 days previously were boosted with a single dose of CoVac^Beta^ (5 μg Beta spike/100 μg AHQ-II) and NAb titers against Beta spike-pseudovirus determined at 1 week post-boost. Data presented as geometric mean ± geometric SD. F. Plasma from mice, rabbits and horses was tested for neutralizing activity against live SARS-CoV-2 infection of Vero E6 cells. The dotted line shows the limit of detection. Significant differences between groups were determined by one-way ANOVA; *, *P* < 0.05; **, *P* < 0.01; ***, *P* < 0.001. Panel A-D created with BioRender.com.

Although CoVac-II immunization affords some level of cross-neutralization against the Beta variant, vaccines currently in use display reduced efficacy against this variant when assessed in placebo-controlled or test-negative-control trials (8, 18, 19). We therefore used our established systems for rapid and high-level production of trimeric viral antigens (10, 20) to produce the Beta spike antigen. Mice vaccinated with CoVac-II 8 months earlier (or control groups) were boosted with a single dose of Beta spike antigen formulated in AHQ-II (CoVac^Beta^). 2 weeks later, the ability of their plasma to neutralize Beta spike-pseudotyped virus was determined (21). The increase of NAbs in response to this booster injection was greatest in mice previously vaccinated with SpK^Alum^ or CoVac-II (approximate increase of 16-fold compared to pre-boost levels); however, responses were maximal in the CoVac-II prime, CoVac^Beta^ boosted group (Fig. 2E). High numbers of spike-specific, multifunctional Th1 CD4^+^ cells were observed in PBMCs from the CoVac-II/CoVac^Beta^ group (Fig. S3). To determine the breadth of cross-neutralization afforded by boosting with the Beta variant, plasma from CoVac-II-primed, CoVac^Beta^-boosted mice was examined for neutralization of ‘wild-type’ virus and three VOCs; Alpha, Beta, and Delta. In pre-boost samples, all VOCs were neutralized; however, titers were reduced approximately 10-fold against Beta compared to ‘wild-type’ virus (Fig. 2F). However, boosting with CoVac^Beta^ resulted in enhanced NAb titers against all VOCs, with greatest increase seen against the Beta variant (Fig. 2F). Notably, neutralization titers against the Delta variant were high (>10^4^) with only a small reduction in titer (approximately 2.2-fold) compared to wild-type virus. Of note, these NAb titers are at least 1 order of magnitude higher than the average human convalescent response, which we have previously assessed with similar methodology, and are comparable with that of ‘elite’ neutralizers (4).

In conclusion, the CoVac-II subunit vaccine we described in this report demonstrates remarkable longevity of immune responses (no decay in NAbs up to 8 months postvaccination in mice) and is highly immunogenic in multiple animal models, including rabbits and horses. The waning of immunity observed in convalescent patients (4) and with current vaccines, coupled with low NAb titers correlating with breakthrough infections (22), suggests that maintenance of humoral immunity will be critical to ensure prolonged vaccine-induced protection against disease. NAbs developed in all immunized species are able to effectively neutralize SARS-CoV-2 variants of concern, which can be augmented by boosting with variant-specific spike vaccines. CoVac-II-immunity compares favorably with other vaccines tested in the same models, that have subsequently shown high-levels of protection in humans (15, 23, 24). The excellent safety profile and immunogenicity demonstrated by a AHQ-II-adjuvanted inactivated SARS-CoV-2 vaccine (13), coupled with our ability to manufacture multiple, high quality antigens at scale (10), suggests that AHQ-II/spike protein combinations could constitute safe, affordable and mass-manufacturable COVID-19 vaccines for global distribution.

## Data availability.

Data supporting the findings of this study are available within the article and as supplementary material. Flow cytometry (FCS) files can be found at https://flowrepository.org/id/FR-FCM-Z4UU and https://flowrepository.org/id/FR-FCM-Z4VY.
