# Supplementary material for: High-Titer Neutralizing Antibodies against the SARS-CoV-2 Delta Variant Induced by Alhydroxyquim-II-Adjuvanted Trimeric Spike Antigens
Source: Microbiol Spectr. 2022 Feb 16;10(1):e01695-21. doi: 10.1128/spectrum.01695-21 (PMC8849074; doi:10.1128/spectrum.01695-21)
Supplement: SUPPLEMENTAL FILE 1 — Supplemental material. Download SPECTRUM01695-21_Supp_1_seq2.pdf, PDF file, 0.6 MB [file spectrum01695-21_supp_1_seq2.pdf]

# SUPPLEMENTARY MATERIAL

# High-titer neutralising antibodies against the SARS-CoV-2 Delta variant induced by Alhydroxyquim-II-adjuvanted trimeric spike antigens

## Supplementary Methods

### Immunisation

Female C57BL/6 mice (6-8 weeks of age) were purchased from Australian BioResources (Moss Vale, Australia), and housed at the Centenary Institute in specific pathogen-free conditions. All mouse experiments were performed according to ethical guidelines as set out by the Sydney Local Health District (SLHD) Animal Ethics and Welfare Committee.

Ancestral (Wuhan) and Beta SARS-CoV-2 full-length spike stabilised, trimeric proteins were expressed in CHO cells and purified as previously described (1). Mice (n=3-5) were vaccinated subcutaneously (s.c) in the footpad once with 5 μg of spike antigen (ancestral) combined with 100 μg of Alhydrogel (Alum; Invivogen, California, USA) or 100 μg Alhydroxiquim-II (Virovax LLC, Kansas, USA) and boosted three weeks after the first vaccination. In some experiments, mice were further boosted with 5 μg B.1.351/Beta spike protein in 100 μg Alhydroxiquim-II. Mice were bled fortnightly after the first immunisation and plasma was collected after centrifugation at 300 x *g* for 10 min. Remaining blood was resuspended in PBS Heparin 20 U/mL, stratified on top of Histopaque 10831 (Sigma-Aldrich, Missouri, USA) and the PBMC layer collected after gradient centrifugation.

Rabbit and horse immunisation experiments were performed at Envigo/Cocalico Biologicals (Reamstown, PA, USA), and at East Tennessee Clinical Research, Inc. (Rockwood, TN, USA),

respectively, in accordance with institutional guidelines. Adult New Zealand White rabbits (n=4 per group) were bled for pre-immune sera via the marginal vein of the pinna, and immunised intramuscularly (i.m) in the flank region on Days 1 and 15 (Prime + One-boost regimen) with 5 μg of spike antigen (ancestral), adjuvanted with 200 μg (Al content) of either Alhydroxiquim-II, or Alhydrogel, each formulated in 0.2 mL saline. Horses (n=3) were immunised i.m. in the cervical region on Days 1 and 15 with 20 μg spike/500 μg Alhydroxiquim-II, or Alhydrogel.

## Flow cytometry assays

To examine T and B cell populations, cells were collected from popliteal lymph nodes 7 days post immunisation and surface stained ($2x10^6$ cells) with Fixable Blue Dead Cell Stain (Life Technologies, California, USA), spike-AF647 (1 μg) and/or the antibodies listed in Supplementary Table 1. Where required, Cells were then fixed and permeabilised using fixation/permeabilisation kit (ThermoFisher, Massachusetts, USA) according to the manufacturer's protocol. To assess spike-specific cytokine induction by T cells, murine PBMCs were stimulated for 4 hrs with spike (5 μg/mL) and then supplemented with Protein Transport Inhibitor cocktail (Life Technologies) for a further 10-12 hrs. Cells were surface stained with Fixable Blue Dead Cell Stain (Life Technologies) and marker-specific fluorochrome-labeled antibodies (Supplementary Table 1). Cells were then fixed and permeabilised using the BD Cytofix/Cytoperm™ kit (Beckton Dickinson, New Jersey, USA) according to the manufacturer's protocol and intracellular staining was performed to detect cytokines IFN-γ, IL-2, TNF, IL-17 (see Supplementary Table 1 for details). All samples were acquired on a BD LSR-Fortessa (BD) or a BD-LSRII and assessed using FlowJo™ analysis software v10.6 (Treestar, Oregon, USA).

## Live SARS-CoV-2 and pseudovirus neutralisation assay

High-content fluorescence microscopy was used to assess the ability of plasma from mice to inhibit live SARS-CoV-2 infection and the resulting cytopathic effect in live permissive cells (VeroE6, final MOI=0.05), using previously described methodology (2). Alpha (B.1.1.7), Beta (B1.351) and Delta (B.1617.2) variants were compared against 'wild-type' D614G virus from the same clade (B.1.319), or Ancestral virus (Wuhan). The cut-off for determining the neutralisation endpoint titer of diluted serum samples was set to ≥50% neutralisation. Neutralising antibody titers in rabbit and horse immune sera were quantified at Virovax using an automated, liquid-handler-assisted, high-throughput, microfocus neutralisation/high-content imaging methods developed at ViroVax. Briefly, serially-diluted sera (paired pre-immune and immune) and SARS-CoV-2 virus (final MOI of 10) were added to $10^6$/mL Vero cells (ATCC® CCL-81) containing 50 μg/mL of propidium iodide (PI) and plates were then loaded in an IncuCyte S3 high-content imaging system (Essen Bioscience/Sartorius, Ann Arbor, Michingan, USA). Longitudinal image acquisition and processing for virus-induced cytopathic effect (CPE) and cell death (PI uptake) were performed every six hours, until cell death profiles had crested and stabilised (3.5 days). Neutralising antibody titers (expressed as $IC_{50}$) were obtained from four-parameter logistic curve-fits of cell death profiles using OriginPro 9 (Origin Lab Corp, Massachusetts, USA).

Replication-deficient SARS-CoV-2 Spike pseudotyped lentivirus particles were generated by co-transfecting GFP-luciferase vector and Ancestral (2) or Beta (3) spike expression constructs with lentivirus packaging and helper plasmids into 293T cells using Fugene HD (Promega, Wisconsin, USA) as previously described (4). To determine neutralising antibody titers, pseudovirus particles were incubated with serially diluted plasma samples at 37°C for 1 hr prior to spinoculation (800xg) of ACE2 over-expressing 293T cells(4). Seventy-two hr post-

transduction, cells were fixed and stained with Hoechst 33342 (NucBlue™ Live ReadyProbes™ Reagent, Invitrogen) as per the manufacturers instruction's, imaged used an Opera Phenix high content screening system (Perkin Elmer, Massachusetts, USA) and the percentage of GFP positive cells was enumerated (Harmony® high-content analysis software, Perkin Elmer).

## Statistical analysis

The significance of differences between experimental groups was evaluated by one-way analysis of variance (ANOVA), with pairwise comparison of multi-grouped data sets achieved using Dunnett's *post-hoc* test. Differences were considered statistically significant when $p \leq 0.05$.


1. Pino P, Kint J, Kiseljak D, Agnolon V, Corradin G, Kajava AV, Rovero P, Dijkman R, den Hartog G, McLellan JS, Byrne PO, Wurm MJ, Wurm FM. 2020. Trimeric SARS-CoV-2 Spike Proteins Produced from CHO Cells in Bioreactors Are High-Quality Antigens. *Processes* 8:1539.
2. Tea F, Ospina Stella A, Aggarwal A, Ross Darley D, Pilli D, Vitale D, Merheb V, Lee FXZ, Cunningham P, Walker GJ, Fichter C, Brown DA, Rawlinson WD, Isaacs SR, Mathivanan V, Hoffmann M, Pohlman S, Mazigi O, Christ D, Rockett RJ, Sintchenko V, Hoad VC, Irving DO, Dore GJ, Gosbell IB, Kelleher AD, Matthews GV, Brilot F, Turville SG. 2021. SARS-CoV-2 neutralizing antibodies: Longevity, breadth, and evasion by emerging viral variants. *PLoS Med* 18:e1003656.
3. Tada T, Dcosta BM, Samanovic MI, Herati RS, Cornelius A, Zhou H, Vaill A, Kazmierski W, Mulligan MJ, Landau NR. 2021. Convalescent-Phase Sera and Vaccine-Elicited Antibodies Largely Maintain Neutralizing Titer against Global SARS-CoV-2 Variant Spikes. *mBio* 12:e0069621.
4. Norman A, Franck C, Christie M, Hawkins PME, Patel K, Ashhurst AS, Aggarwal A, Low JKK, Siddiquee R, Ashley CL, Steain M, Triccas JA, Turville S, Mackay JP, Passioura T,

Payne RJ. 2021. Discovery of Cyclic Peptide Ligands to the SARS-CoV-2 Spike Protein Using mRNA Display. *ACS Cent Sci* 7:1001-1008.

**Supplementary Table 1: Flow cytometry antibodies used in this study**

**T cell panel**

| **Marker** | **Fluorophor** | **Location** | **Clone** | **Dilutio** | **Company** | **Cat. #** |
|---|---|---|---|---|---|---|
| Live/Dead | Blue | Surface | | 1:300 | ThermoFisher | L2310 |
| Fc Block | purified | Surface | 2462 | 1:300 | BD | 553141 |
| CD4 | AF700 | Surface | RM414 | 1:200 | BD | 557956 |
| CD44 | BV605 | Surface | IM7 | 1:300 | BD | 563058 |
| CD8 | APC-Cy7 | Surface | 53-6.7 | 1:200 | BD | 557654 |
| CXCR5 | Biotin | Surface | 2G8 | 1:100 | BD | 551960 |
| PD-1 | BV711 | Surface | 29F.1A1 | 1:200 | Biolegend | 135231 |
| Streptavidi | PE-Cy7 | Surface | | 1:200 | BD | 557598 |
| BCL-6 | AF647 | Intracellula | K112-91 | 1:200 | BD | 561525 |
| CD19 | BV785 | Surface | 1D3 | 1:200 | BD | 563333 |
| IFN-γ | PECy7 | Intracellula | XMG1-2 | 1:200 | BD | 557649 |
| IL-17 | PB | Intracellula | TC11- | 1:200 | BioLegend | 506918 |
| IL-2 | PE | Intracellula | JES6- | 1:200 | BD | 554428 |
| TNF | PerCP- | Intracellula | MP6- | 1:200 | BD | 560659 |

**B cell panel**

| **Marker** | **Fluorophore** | **Location** | **Clone** | **Dilution** | **Company** | **Cat. #** |
|---|---|---|---|---|---|---|
| Live/Dead | Blue | Surface | | 1:300 | ThermoFisher | L23105 |
| Fc Block | purified | Surface | 2462 | 1:300 | BD | 553141 |
| CD19 | BV785 | Surface | 1D3 | 1:200 | BD | 563333 |
| Spike | AF647 | Surface | | 1:50 | | |
| GL7 | AF488 | Surface | GL7 | 1:200 | Biolegend | 144612 |
| MHCII | AF700 | Surface | M5/114.15.2 | 1:200 | Biolegend | 107622 |
| CD138 | BV605 | Surface | 281-2 | 1:200 | Biolegend | 142516 |
| CD38 | APC-Cy7 | Surface | 90 | 1:200 | Biolegend | 102728 |
| IgM | BV421 | Surface | RMM-1 | 1:200 | Biolegend | 406518 |
| IgD | PerCP5.5 | Surface | 11-26c.2a | 1:200 | BD | 564273 |

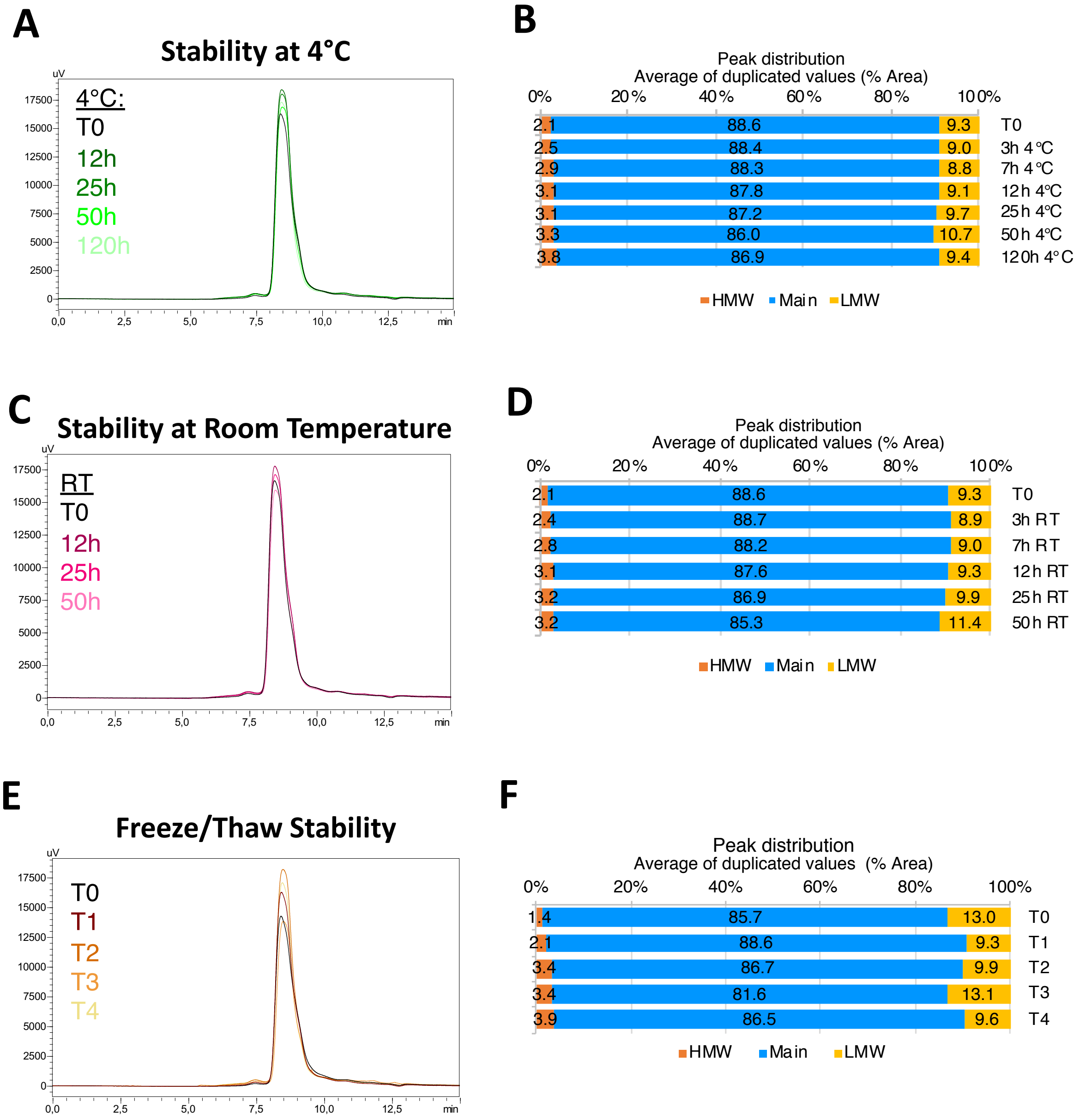


**Supplementary Figure 1.** Stability of trimeric spike protein produced in CHO cells. Chromatographs (**A, C, E**) and peak distribution (**B, D, F**) of spike protein after storage at 4ºC (**A, B**) and room temperature (RT, **C, D**) or after 4 cycles of freeze-thawing (**E, F**).

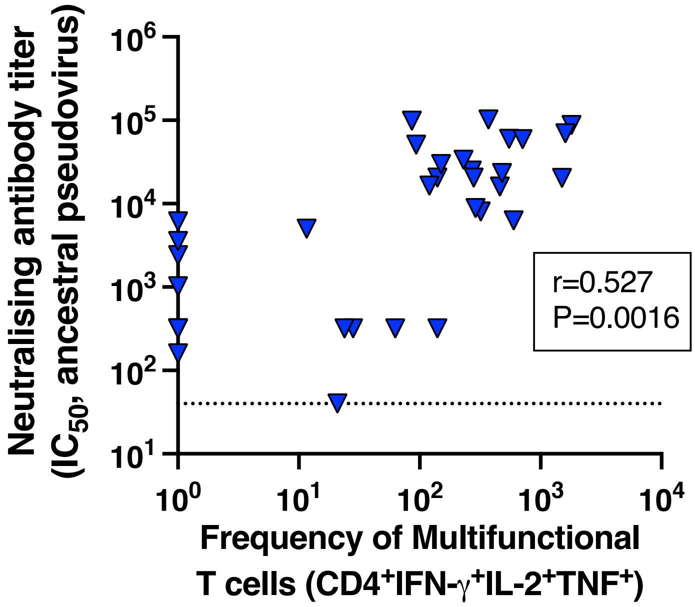


**Supplementary Figure 2.** Spearman correlation of neutralizing antibody titer and multifunctional $CD4^+$ T cells after vaccination with AHQ-adjuvanted spike vaccines. The dotted line shows the limit of detection.

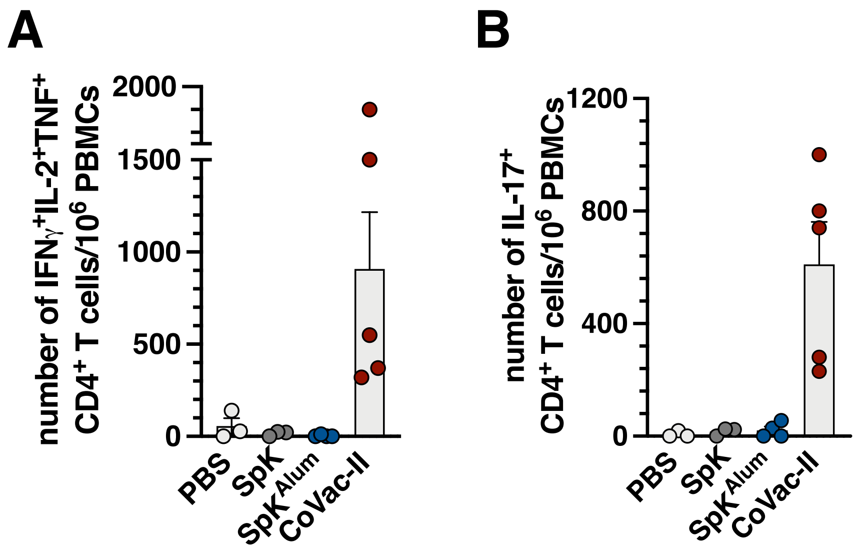


**Supplementary Figure 3.** Mice vaccinated 252 days previously were boosted with a single dose of CoVac$^{Beta}$ (5 µg Beta spike/100 µg Alhydroxiquim-II). PBMCs isolated one week post-boost were restimulated *ex vivo* with 5 µg/mL of Beta spike and the number of circulating CD4$^{+}$ expressing IFN-γ, IL-2 and TNF (**A**) or IL-17 (**B**) determined by flow cytometry.
